# The anti-inflammatory and antioxidant effects of Montelukast on lung sepsis in adult mice

**DOI:** 10.25122/jml-2021-0269

**Published:** 2022-06

**Authors:** Zainab Ali Alnfakh, Dhefaf Hameed Al-Mudhafar, Rana Talib Al-Nafakh, Abdullah Elttayef Jasim, Najah Raiesh Hadi

**Affiliations:** 1 Department of Pharmacology & Therapeutics, Faculty of Medicine, University of Kufa, Kufa, Iraq; 2 Middle Euphrates Unit for Cancer Researches, Faculty of Medicine, University of Kufa, Kufa, Iraq; 3 College of Medicine, Iraqi University, Baghdad, Iraq

**Keywords:** Montelukast, IL-6, IL-1B, IL-17, F2 isoprostane, sepsis, ARDS – Acute respiratory distress syndrome, ALI – Acute lung injury, ANOVA – Analysis Of Variance, C° – Celsius Degree, CLP – Cecal Ligation And Puncture, DMSO – Dimethyl Sulfoxide, IL-1β – Interleukin-1beta, IL-6 – Interleukin 6, IL-17 – Interleukin 17, IP – Intraperitoneal

## Abstract

One of the most complex clinical challenges facing medical practice is sepsis-induced lung dysfunction resulting from polymicrobial sepsis. Although many therapeutic approaches have been used in such clinical challenges, there is still further need for a new effective therapeutic approach. The objective of this study was to investigate if Montelukast could protect the lungs during polymicrobial sepsis by regulating inflammatory markers and the oxidative stress pathways. Twenty-four mature male Swiss-albino mice aged 8–12 weeks, with a weight of 20–30 g, were randomized into 4 equal groups (n=6), sham (laparotomy without cecal ligation and puncture (CLP)), CLP (laparotomy with CLP), vehicle 1 (equivalent volume of DMSO 1 hour prior to CLP), Montelukast (10 mg/kg IP 1 hour prior to CLP). Lung tissue pro-inflammatory mediators IL-6, IL-1β, IL-17, LTB-4 12(S) HETE, and oxidative stress were assessed using ELISA. The levels of F2 isoprostane were considerably greater in the sepsis group (p<0.05) as compared to the sham group, while Montelukast was significantly lower (p<0.05) in these inflammatory mediators and oxidative stress as compared to the sepsis group. Histologically, the lung tissue damage was significant (p<0.05) in all mice in the sepsis group, while Montelukast significantly reduced lung tissue injury (p<0.05). The current findings indicated that Montelukast could attenuate lung dysfunction during CLP-induced polymicrobial sepsis in male mice through their modulating effects on pro-inflammatory and oxidative stress downstream signalling pathways and subsequently decrease lung tissue cytokine concentrations (IL-1β, IL-6, IL-17, LTB-4, and 12(S)HETE).

## INTRODUCTION

The most prevalent condition among patients treated in critical care units is sepsis, an infection-related systemic inflammatory response syndrome [1]. Sepsis, usually called septic shock, is a pathological condition marked by severe hypotension, increased metabolic acidosis, systemic inflammatory response syndrome (SIRS), multiple organ failure syndrome, tissue damage, acute respiratory distress syndrome (ARDS), acute limb ischemia (ALI), and mortality [2]. Increased endothelial vascular permeability begins during sepsis, and plasma extravasates in several organs, resulting in bacterial proliferation, which may contribute to severe tissue damage [3]. The discovery of key products such as endotoxin, cytokines, and arachidonic acid metabolism products, as well as discovering that integrating these molecules into human volunteers or laboratory animals could produce a clinical syndrome similar to sepsis, fuelled a search for specific agents to inhibit, block, neutralize, or limit the potential distributive effects [4]. Monocytes control the innate immune response to microorganisms by producing inflammatory cytokines such as interleukin-6 (IL-6), IL-17, IL-1β, and SIRS, mortality and multiple organ failure are all possible outcomes [5]. High amounts of these pro-inflammatory cytokines contribute to the development of endotoxin shock [6]. For instance, IL-17 is linked to neutrophil recruitment, IL-6 is linked to tissue damage caused by inflammation, and IL-1B is linked to pulmonary inflammation and emphysema [7]. Although IL-17, IL-6, and IL-1B are important in aseptic shock, the exact processes by which they are produced are unknown.

## MATERIAL AND METHODS

The research was conducted at the Faculty of Medicine/University of Kufa, Department of Pharmacology and Therapeutics, and the Middle Euphrates Unit for Cancer Researches.

### Study design

Twenty-four adult male albino Swiss mice, weighing 20–30 g and aged 8–12 weeks, were obtained from the Animal Resources Centre of the College of Science at the University of Kufa. The animals were kept in the animal house at the University of Kufa, at a temperature of 25°C and humidity of 60–65 percent, with a 12 hours light: 12 hours dark cycle. The mice in this study were randomly allocated into four groups, each with six mice, as follows:

- Sham group: All of the animals in this group were given anaesthesia and laparotomy surgery but without cecal ligation and puncture (CLP);
- Cecal ligation and puncture operated group (CLP) (sepsis group): All mice in this group had their cecum ligated and punctured;
- Vehicle group: 1 hour before being treated with CLP, all animals were given an equal volume of dimethyl sulfoxide (DMSO) intraperitoneal (IP) injection;
- Montelukast pre-treated group: 1 hour before CLP, all mice in this group were given a 10 mg/kg Montelukast IP injection.

### Experimental procedure

Mice were numbed intraperitoneally with 100 mg/kg ketamine and 10 mg xylazine [8]. An abdominal arthroscopy was conducted through a 1.5 cm midline incision, revealing the cecum. After that, the cecum was ligated just below the ileocecal valve and pierced twice with a G-20 needle before being restored to its normal position. Following this, a 5/0 surgical stitch was used to close the abdomen. Mice were checked for various indicators of illness every four hours for 24 hours before being returned to their cages with unlimited food and drink. The surgical control group consisted of sham surgically operated mice (anaesthesia and laparotomy without CLP) [9].

### Preparation of drugs

#### Montelukast

Montelukast sodium was procured from Chemscene in the United States and prepared in 50 mg/ml DMSO before being administered IP at a dose of 10 mg/kg.

### Tissue processing for the assessment of IL-6, IL-1, IL-17, LTB-4, 12(S) HETE, and F2-isoprostane

To remove any blood, a cold isotonic sodium chloride solution was used to cleanse the lung (0.9 percent) and preserved in a deep freeze at -80 degrees Celsius. The lung piece was then homogenized in 1:10 W/V phosphate-buffered saline with 1% Triton X-100 and 1% protease inhibitor cocktail using a high-intensity ultrasonic liquid processor [10]. Next, the homogenized lung piece was subjected to a slightly increased ultrasonic fluid processor for optimum homogenization, which resulted in further disruption of the cell membranes. The homogenates were centrifuged for 20 minutes at 4°C at 3000 rpm, and the residue was utilized to measure the levels of IL-6, IL-1, IL-17, LTB4, 12(S) HETE, and F2-isoprostane using Elisa kits (Elabscience Technology Laboratory).

### Preparation of tissue samples for histopathology

Lung tissues from mice sacrificed were rinsed in a cool isotonic NaCl solution of 0.9 percent to eliminate red blood cells and clots, then placed in 10% formalin and treated in paraffin tissue blocks, where microscopic slices of 5 m thickness were collected and stained with hematoxylin-eosin dye [11]. Following the fixation of the specimens, the first stage was dehydration, which involved immersing the specimens in ethanol for two hours for each concentration (70, 80, 90, and 100 percent) to extrude any leftover formalin or H_2_O from the samples. Then they were cleaned with xylene (an organic solvent), which was used to remove the alcohol from the samples in order to seep with paraffin wax. The histopathology test was performed at original magnifications of X100 [12] and scored by the percentage of tissue damage as follows:

**Score 0:** no damage, normal architecture;

**Score 1 (mild):** less than 25% damage;

**Score 2 (moderate):** 25–50% damage;

**Score 3 (severe):** 50–75% damage;

**Score 4 (highly severe):** 75–100% damage.

### Statistical Analysis

SPSS version 26 was used for statistical analysis. The data was provided as a Mean SEM. For comparisons of different groups, ANOVA (analysis of variance) was employed. After that, a post-hoc test with Bonferroni correction was performed. The Kruskal-Wallis test was used to determine the statistical significance of the difference in mean scores for histological abnormalities of lung tissue across several groups. P values less than 0.05 were deemed statistically significant in all tests.

## RESULTS

### Montelukast decreased inflammatory IL-6, IL-1β, and IL-17 markers in lung tissue

**Figure 1:**
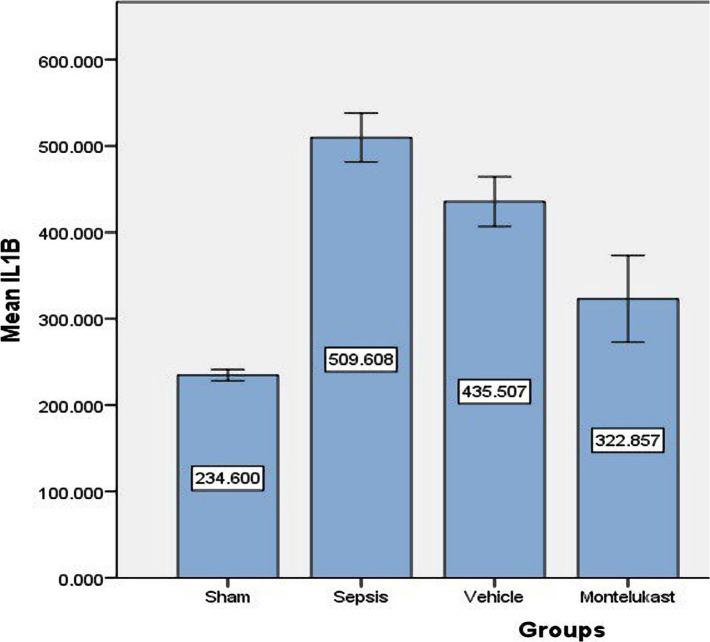
Mean lung tissue level of IL-17 (pg/ml)±SEM of the four groups at the completion of the experiment; sham vs. sepsis group (p-value=0.00001), Montelukast vs. sepsis and vehicle group (p-value=0.252).

**Figure 2:**
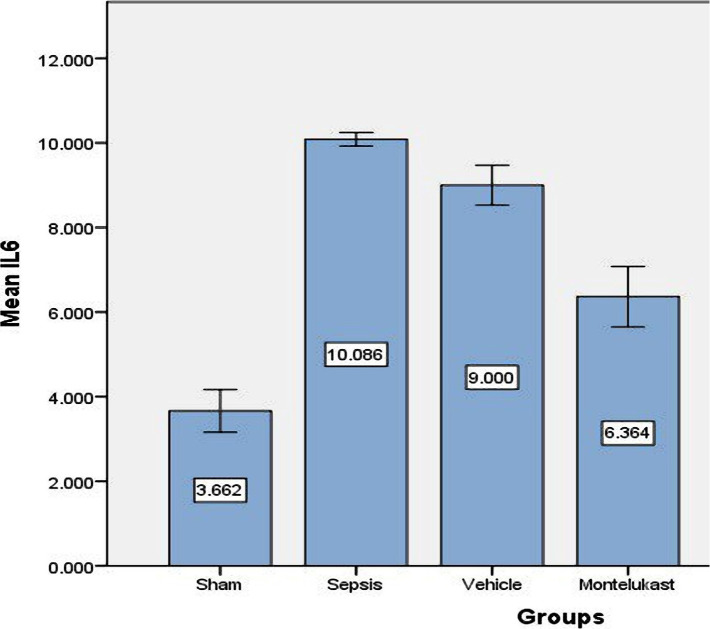
Lung tissue slope of IL-6 (pg/ml)±SEM of the four experimental groups at the completion of the experiment; sham vs. sepsis group (p-value=0.00001), Montelukast vs. sepsis and vehicle group (p-value=0.00001).

**Figure 3:**
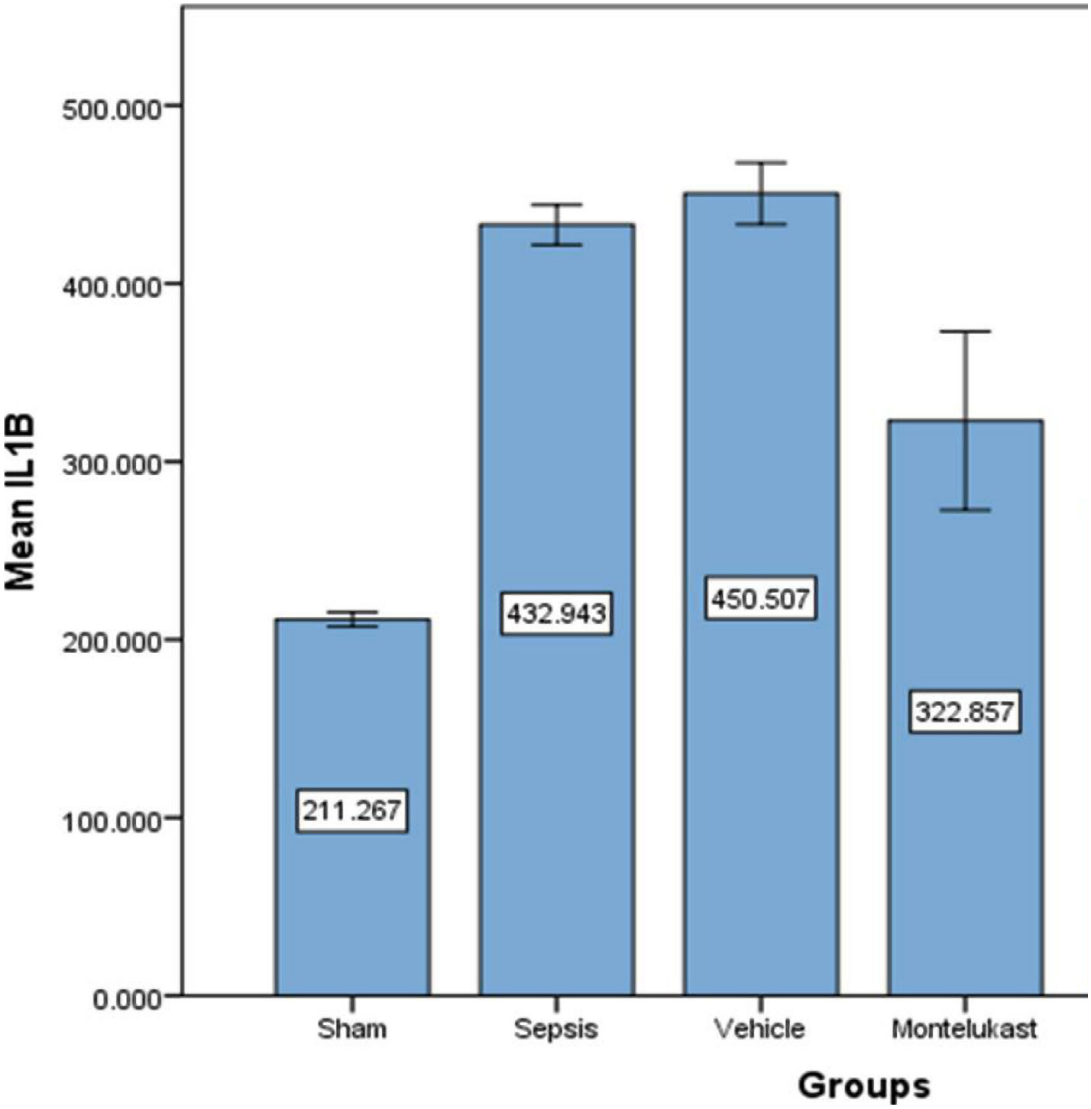
Lung tissue slope of IL-1B (pg/ml)±SEM of the four experimental groups at the completion of the experiment; sham vs. sepsis group (p-value=0.00001), Montelukast vs. sepsis and vehicle group (p-value=0.00001).

When comparing the sepsis group to the sham group, the lung level of IL-17 was considerably (p<0.05) higher in the sepsis (control) group, and the difference between the sepsis and vehicle groups was negligible. When comparing sepsis and vehicle groups, lung levels of IL-17 in the Montelukast treated group was slightly lower (Figure 1). The levels of IL-6 and IL-1B in lung tissue were significantly (p<0.05) increased in the sepsis group compared to the sham group. When contrasting with the sepsis group, the 10 mg/kg Montelukast pre-treated group showed considerably lower expression of inflammatory markers IL-6 and IL-1B (Figures 2 and 3).

### The effect of Montelukast treatment on LTB4

**Figure 4:**
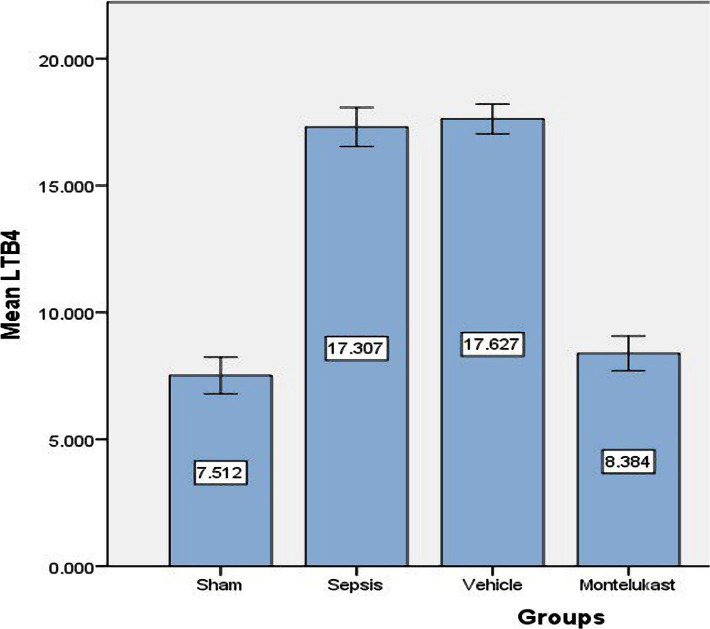
Mean lung tissue level of LTB-4(pg/ml)±SEM of the four experimental groups at the end of the experiment; sham vs. sepsis group (p-value=0.00001), Montelukast vs. sepsis and vehicle group (p-value=0.00001).

For more documented and evident results, we examined the tissue level of the specific lung leukotrienes marker LTB4, 24 hours after polymicrobial sepsis induced by the CLP model, in all experimental groups using ELISA assay protocol. When comparing sepsis and vehicle groups to sham groups, ELISA results revealed that sepsis and vehicle groups had significantly greater tissue levels (p<0.05). Compared to sepsis and the vehicle group, the Montelukast pre-treated groups had significantly lower LTB4 levels (p<0.05). Changes in the lung slope of the LTB4 level can be seen in Figure 4.

### The effect of Montelukast treatment on 12(S) HETE

**Figure 5:**
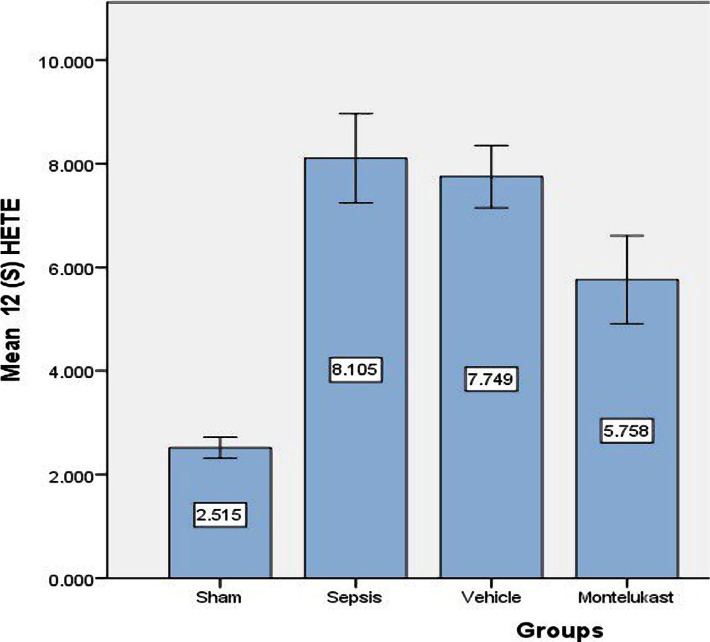
Lung tissue level of 12(S) HETE (ng/ml)±SEM of the four experimental groups at completion of the test; sham vs. sepsis group (p-value=0.00001), Montelukast vs. sepsis and vehicle group (p-value=0.00001).

Furthermore, we investigated the effects of Montelukast treatment on the intracellular signalling pathway. We focused on modulating the effects of our treatment on 12(S) HETE signalling cascades during the polymicrobial sepsis-induced CLP model through ELISA analysis. The 12(S)-HETE expression in lung cells had significant (p<0.05) higher levels in sepsis and vehicles groups as compared with the sham group, while the 12(S)-HETE expression levels were significantly (p<0.05) lower in Montelukast pre-treated groups as compared with sepsis group. Variations in lung levels of 12(S) HETE level are summarized in Figure 5.

### Montelukast decreases oxidative stress (F2-isoprostane) in lung tissue

**Figure 6:**
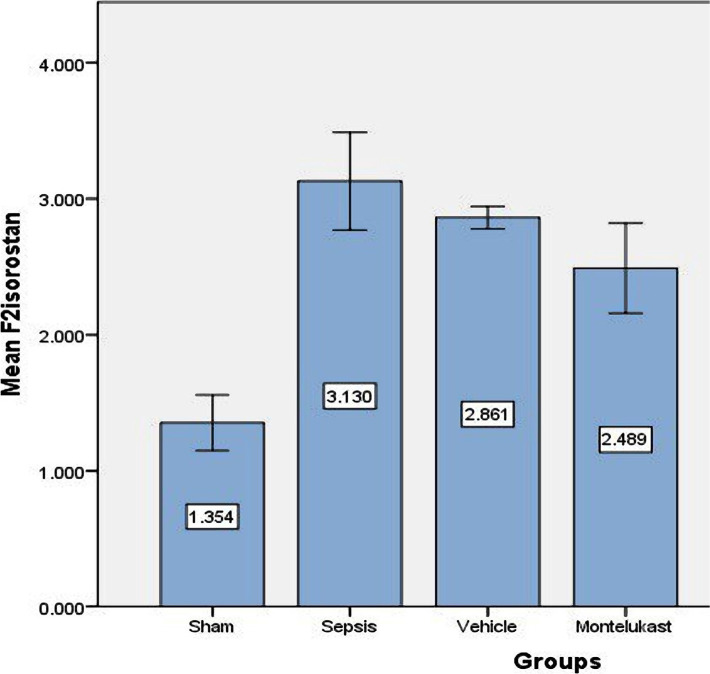
Tissue slope of F2 isoprostane (pg/ml) of the four test groups at the completion of the experiment; sham vs. sepsis group (p-value=0.00001), Montelukast vs. sepsis group (p-value=0.00001).

In contrast to the sham group, mice in the sepsis group had a substantial rise in F2-isoprostane slope in lung tissue. Compared to the sepsis group, pre-treatment with Montelukast reduced the amount of F2-isoprostane in lung tissue (Figure 6).

### Montelukast minimized lung injury

**Figure 7:**
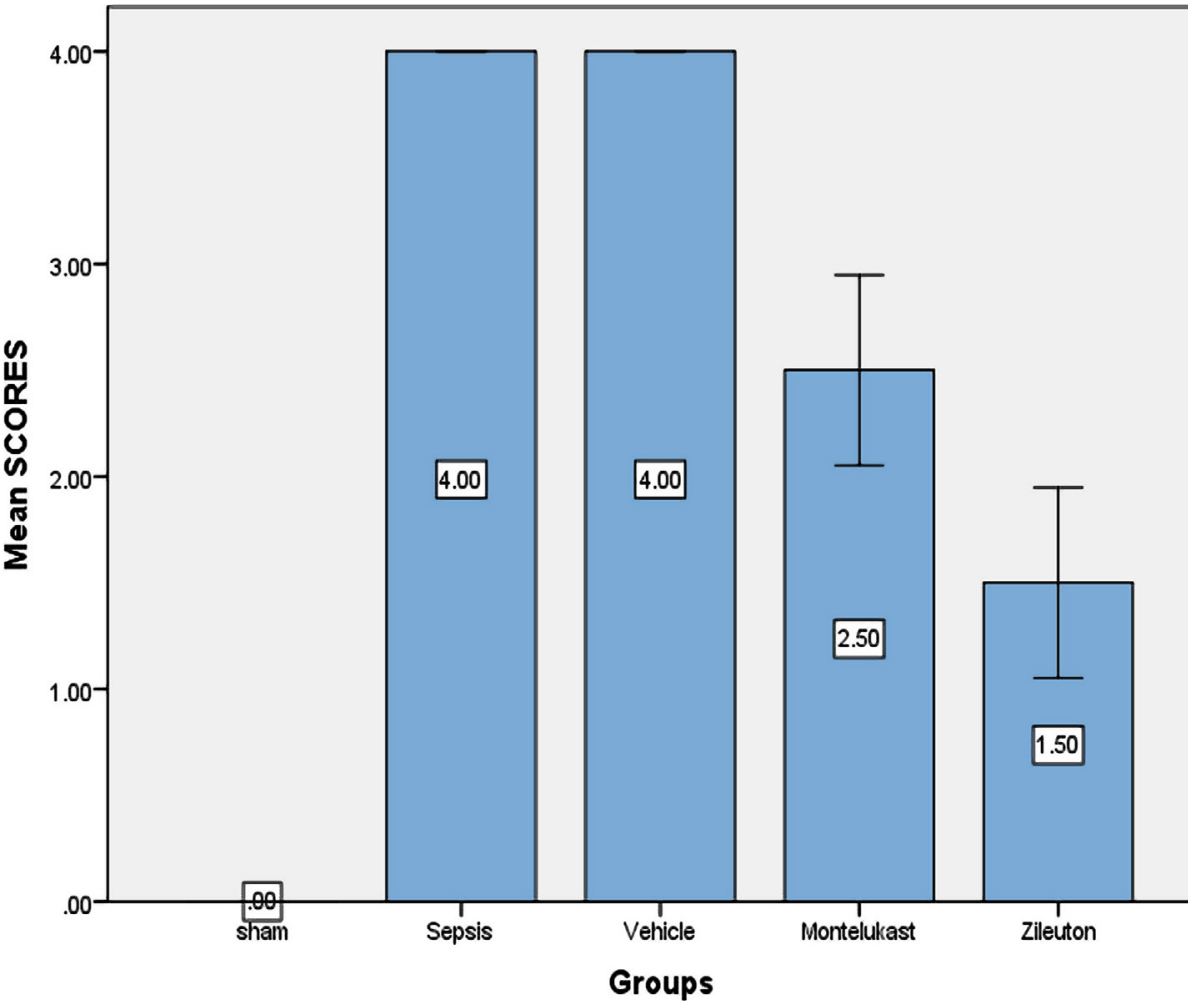
Histopathological result of lung tissue of the four test groups at the completion of the experiment; sham vs. sepsis and vehicle group (p-value=0.0001), Montelukast vs. sepsis group (p-value=0.0001), Montelukast vs. sepsis group, (p-value=0.0001).

**Figure 8:**
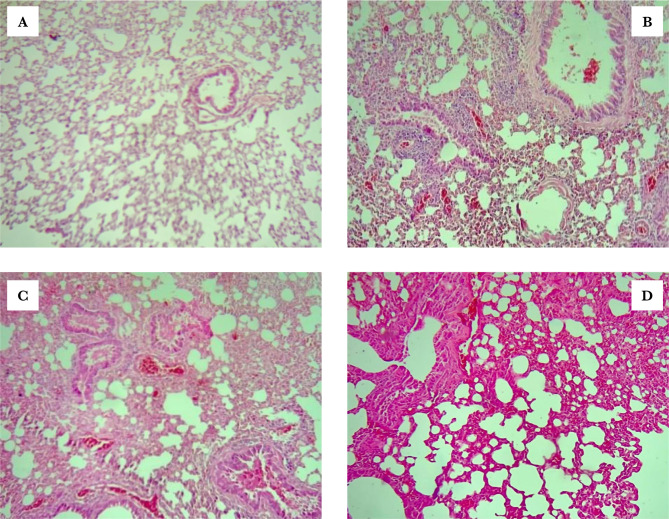
Lung segment for histopathological investigation, spotted with haematoxylin and eosin (x100). A – The sham group's lung segment photomicrograph shows normal lung tissue, no inflammation, no oedema, and no swelling. B – A microscopic examination of a lung slice from the sepsis group shows vascular congestion with perivascular infiltration of mononuclear inflammatory cells. C – The lung segment photomicrograph of the vehicle group shows significant mixed inflammation, vascular congestion, and interstitial edema. D – A microscopic examination of the lung portion from the Montelukast group shows moderate-to-severe inflammation affecting about 40% of the analysed lung tissue.

Histopathological examination showed normal lung tissue in the sham group, in sepsis and vehicle groups, development of congestion, significant perivascular inflammation of mononuclear (lymphocytes, plasma cells) inflammatory cells, interstitial oedema, and localized extravasations of red blood cells. The histological features of the Montelukast pre-treated mice group showed moderate architectural alterations when compared with the sham group (Figure 7 and Figure 8 A–D).

## DISCUSSION

Sepsis is a disorder that causes organ failure due to a faulty host response to infection, which results in an out-of-control inflammatory response and immunological suppression [13]. Severe sepsis still has a significant fatality rate (22–65%) despite advancements in critical care. A significant consequence of sepsis is gradually decreased lung function and susceptibility to intrapulmonary infection among multiorgan failure. In these settings, acute respiratory distress syndrome (ARDS) is a common consequence [14, 15]. The intensity of the inflammatory response and its persistent WBCs activation may dictate the pathologic development of ARDS due to the loss of alveolar compartmentalization and the way that pro-inflammatory cascade activation can reach the bloodstream and cause multiple organ dysfunction syndrome. The cytokines, especially IL-1B, IL-6, and IL-17, are important in lung dysfunction [3]. The research on the role of pharmacological treatments in these instances is yet limited. In this work, the protective impact of Montelukast on improving lung function in mice after poly microbial sepsis was generated using the CLP model.

### Effect of sepsis on pro-inflammatory cytokines (IL1B, IL-6, and IL-17)

Our research showed significantly higher lung tissue levels of pro-inflammatory cytokines (IL1B, IL-6, and IL-17) in sepsis and vehicles groups compared with the sham group, corresponding to other findings [16]. Furthermore, endotoxin shock could cause a significant increase in serum IL-1B, IL-6, and IL-17 [3]. When the immune system responds to the invasion of extracellular infections, it is linked to excessive tissue injury. IL-17 increases the synthesis of IL-6, granulocyte, IL-1B, and prostaglandins in a range of cell types, including fibroblasts, endothelial cells, epithelial cells, keratinocytes, and macrophages [17]. The up-regulation of inflammatory responses is dependent on the production of these cytokines [18]. Increased amounts of pro-inflammatory cytokines can potentially cause microcirculation problems [19]. The hyperpermeability of the pulmonary microvasculature is a major damage produced by sepsis in the progress of ARDS, and therefore alveoli are filled with plasma exudates during this severe systemic inflammation [20]. Furthermore, elevated exudates in alveolar spaces promote alveolar oedema, which occurs in combination with alveolar epithelial cell loss caused by sepsis-induced apoptosis and necrosis. The initial and acute phases of ARDS are characterized by diffuse damage. The capacity for oxygen exchange is diminished due to vascular leakage, alveolar epithelial damage, and the subsequent build-up of free fluid, leading to acute respiratory failure, which develops ARDS, and finally, complete lung function failure and death [21].

### Effect of Montelukast on pro-inflammatory cytokines (IL-1B, IL-6, and IL-17)

Compared to sepsis and vehicle groups, Montelukast pre-treatment groups had significantly reduced lung tissue levels of pro-inflammatory cytokines (IL-1B, IL-6, and IL-17). Following induced CLP, [22] Montelukast therapy substantially reduced blood pro-inflammatory IL-6 cytokine levels. These findings show that the capacity of Montelukast to cause mice to generate less inflammatory cytokines in response to CLP-induced sepsis may reduce cytokine-related organ damage. The anti-inflammatory action of Montelukast in CLP-induced sepsis appears to be due to the inhibition of several pro-inflammatory mediators generated by leukocytes and macrophages. Montelukast inhibited IL-6 production in both [23, 24]. The pro-inflammatory cytokines IL-1B and IL-6, on the other hand, are reduced in Sarcoidosis exosomes, whereas asthma treatment encourages monocytes to produce pro-inflammatory cytokines and CCL2 [25]. In mycoplasma pneumonia, Montelukast lowered serum IL-6, IL-17, and peripheral blood Th1 and Th17 levels [26].

### Effect of sepsis on pro-inflammatory cytokines leukotrienes B4 (LTB4)

Our study showed significantly higher lung tissue levels of LB4 in sepsis and vehicles groups compared with the sham group suggesting a significant increase in 5-LOX activity, corresponding with other findings [27]. Leukotrienes B4 (LTB4) is a potent pro-inflammatory lipid mediator derived from arachidonic acid by 5-Lipoxygenases (5-LO) [28]. The attraction of leukocytes, especially neutrophils, is one of LTB4's main functions [29]. Elevated levels of pro-inflammatory lipid leukotrienes B4 can mediate pro-inflammatory cytokines IL- 1B, 1L-6, and IL-17, key cytokines for the development of endotoxin shock, by leukotrienes B4 receptors (BLT1\2) mediated pathways [3]. The primary neutrophil chemotactic factor secreted by alveolar macrophages is leukotrienes B4. So, high levels of LTB4 during polymicrobial sepsis indicated a risk of lung injury and pulmonary complications [30].

### Effect of Montelukast on pro-inflammatory leukotriene B4

The present study showed that Montelukast pre-treated groups had significantly lower lung tissue levels of LTB4 compared with sepsis and vehicles groups. Montelukast effectively reduced both LPS-induced lung inflammations in a rat model of ARDS and LPS-challenged neutrophils, according to previous research [30]. On the other hand, another study [31] showed a significant decrease in LTB4 levels. Previous studies [32] confirmed that the level of LTB4 analysed using specific ELISA kits significantly increased in peritoneal lavage fluid after LPS-induced endotoxin shock. These findings offered more evidence of the lung-protective effect of Montelukast during polymicrobial sepsis induced by CLP.

### Effect of sepsis on 12(S)-hydroxy eicosatetraenoic acid (12(S) HETE)

There were significantly higher lung tissue levels of 12(S) HETE in the sepsis and vehicles groups compared to the sham group. Similar results were obtained in another study where significantly increased serum 12(S)HETE level in lipopolysaccharide (LPS) induced endotoxin shock [3]. CLP-induced polymicrobial sepsis can produce inflammation of the lungs in its early stages. Immune cell penetration, mucus development, vascular infiltration into the airways, and epithelial cell death are all consequences. As a result, uncontrolled inflammations are at the root of many chronic obstructive lung illnesses, including fibrosis [33]. Increased levels of eicosanoid 12 (S) HETE were observed in the lungs in response to inflammatory triggers [34], and the leakage of neutrophils into the pulmonary space is a defining characteristic of lung damage. One explanation for the link between 12 (S) HETE and lung injury is that in a mouse model of acute lung injury (ALI), CLP-induced sepsis caused inflammation, increased vascular permeability, and upregulation of lipoxygenases. As a result, the production of lipoxygenases in alveolar macrophages and fibroblasts leads to bronchial epithelial injury via a 12(S) HETE-dependent mechanism involving leukotrienes.

### Effect of Montelukast on 12(S)-hydroxy eicosatetraenoic acid (12(S) HETE)

The present investigation found that compared to sepsis and the vehicle group, the Montelukast-pre-treated group had reduced lung tissue levels of 12(S) HETE. To our knowledge, there may be no prior investigation on the effect of Montelukast on the 12(S) HETE expression pathway in lung sepsis.

### Effect of sepsis on oxidative stress marker (F2 isoprostane)

This research discovered that the F2 isoprostane slope in the sepsis group was considerably higher than in the sham group. Increased oxidative stress and the production of reactive oxygen species are crucial in initiating and maintaining the inflammatory response. The measurement of F2-isoprostanes is one of the consistent processes for measuring oxidative stress conditions [35]. The outcomes within the present study agree with the ones which showed that plasma F2-isoprostane levels accelerated [36].

### Effect of Montelukast on oxidative stress marker (F2 isoprostane)

The existing study confirmed that the lung level of oxidative stress in the Montelukast treated group was lower than in the sepsis and vehicle groups [37]. Montelukast monotherapy showed only a minor but not statistically significant improvement in all oxidative stress and DNA damage parameters in children with asthma [37]. To our knowledge, no previous research has been done on the effect of Montelukast on F2-isoprostane in sepsis.

## CONCLUSIONS

Our findings revealed that Montelukast could attenuate lung dysfunction during CLP-induced polymicrobial sepsis in male mice through their modulating effects on pro-inflammatory and oxidative stress downstream signalling pathways and subsequently decreased lung tissue levels of cytokines (IL-1β, IL-6, IL-17, LTB-4 and 12(S) HETE).
